# All–Inorganic Perovskite Quantum Dot–Based Blue Light–Emitting Diodes: Recent Advances and Strategies

**DOI:** 10.3390/nano12244372

**Published:** 2022-12-08

**Authors:** Yuyu Hu, Shijie Cao, Peng Qiu, Meina Yu, Huiyun Wei

**Affiliations:** 1 Beijing Advanced Innovation Center for Materials Genome Engineering, Beijing Key Laboratory for Magneto–Photoelectrical Composite and Interface Science, School of Mathematics and Physics, University of Science and Technology Beijing, Beijing 100083, China; 2 Institute for Advanced Materials and Technology, University of Science and Technology Beijing, Beijing 100083, China; 3 Engineering Research Center of Clinical Functional Materials and Diagnosis & Treatment Devices of Zhejiang Province, Wenzhou Institute, Wenzhou Institute of Biomaterials & Engineering, University of Chinese Academy of Sciences, Wenzhou 325027, China

**Keywords:** all–inorganic lead halide perovskite, quantum dot, blue emission, light–emitting diodes

## Abstract

Light–emitting diodes (LEDs) based on all–inorganic lead halide perovskite quantum dots (PQDs) have undergone rapid development especially in the past five years, and external quantum efficiencies (EQEs) of the corresponding green– and red–emitting devices have exceeded 23%. However, the blue–emitting devices are facing greater challenges than their counterparts, and their poor luminous efficiency has hindered the display application of PQD–based LEDs (PeQLEDs). This review focuses on the key challenges of blue–emitting PeQLEDs including low EQEs, short operating lifetime, and spectral instability, and discusses the essential mechanism by referring to the latest research. We then systematically summarize the development of preparation methods of blue emission PQDs, as well as the current strategies on alleviating the poor device performance involved in composition engineering, ligand engineering, surface/interface engineering, and device structural engineering. Ultimately, suggestions and outlooks are proposed around the major challenges and future research direction of blue PeQLEDs.

## 1. Introduction

All–inorganic cesium lead halide CsPbX_3_ (X = Cl, Br, I) perovskites have attractive photophysical properties, including excellent color purity, narrow emission bandwidth, high photoluminescence quantum yields (PLQYs), and easily tunable emission band, showing great potential in LED applications [1]. In the past few years, great progress in the performance of perovskite LEDs (PeLEDs) has been made. The EQEs of red– and green–emitting PeLEDs have exceeded 20%, while the development of blue–emitting PeLEDs still lags far behind their counterparts. For blue PeLEDs, the current poor brightness and short operating lifetime still do not meet the requirements of the National Television System Committee (NTSC), which seriously hampers the development of full–color PeLEDs. It is well known that the light emission of perovskites can be tailored by adjusting their composition and dimensions. Dimensionality engineering is effective for promoting a blueshift of light emission. Two–dimensional or quasi–2D perovskite layers and 0D PQDs with quantum confinement effects have been applied to blue PeLEDs [2,3,4,5]. This review will focus on the development of bluevemitting CsPbX_3_–based PeQLEDs.

**Figure 1:**
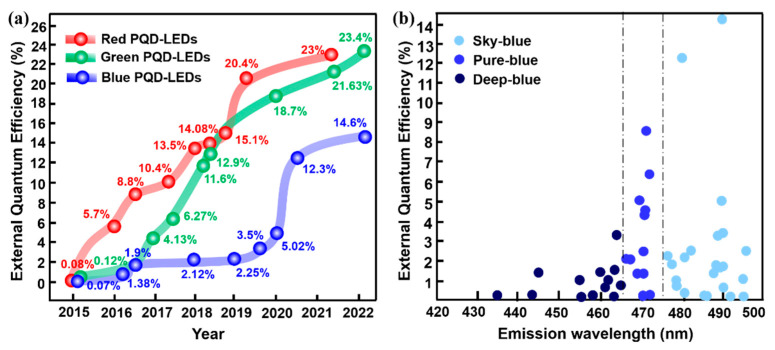
(**a**) Progress diagram of the EQEs for the reported PeQLEDs; (**b**) EQEs of the reported sky–, pure–, and deep–blue PeQLEDs.

In 2015, Song and co–workers [6], for the first time, reported the CsPbX_3_ blue PeQLEDs, exhibiting luminance of 742 cd/m^2^ and EQE of 0.07% at the emission wavelength of 455 nm. Subsequently, the device efficiency experienced a slow growth until 2021 when an EQE of 12.3% was reported. However, as shown in Figure 1, PL emission wavelength of the devices with relatively higher efficiency is mainly concentrated in the sky–blue region (475–495 nm). It is also highly desirable to develop devices with emission less than 470 nm to meet the NTSC standard, namely, the so–called pure–blue (465–475 nm) and deep–blue (420–465 nm) devices [7]. To our knowledge, the EQEs of pure– and deep–blue devices are still less than 10% and 5%, respectively. The development of pure– and deep–blue PeQLEDs is even more challenging since PQDs consisting of mixed Br/Cl composition are usually needed in which halogen separation would lead to the drop of EQE and spectral shift. To date, pure– and deep–blue PeQLEDs exhibit poor brightness and short operating half–life, which seriously hinder their application in display technology. Therefore, designing highly efficient blue PeQLEDs that meet the NTSC standard remains an unresolved challenge. The major problems for blue PeQLEDs could be summarized as considerably lower EQE, efficiency roll–off, and short operating lifetime. To date, various efforts have been performed to deepen understanding for the essential mechanism of the above problems as well as to alleviate the poor device performance. A timely overview of current strategies on blue PeQLEDs and the discussion of the referential approaches for other types of PeLEDs are meaningful to facilitate the further development of high–performance blue PeQLEDs. In this review, we firstly present the main preparation and property regulation methods for blue emission CsPbX_3_ PQDs. Then, the latest research progress of blue PeQLEDs will be systematically summarized, and the corresponding strategies are specifically divided into composition engineering, ligand engineering, surface/interface engineering, and device structural engineering. Ultimately, suggestions and outlooks are proposed, aiming at the major challenges and future development of blue PeQLEDs.

## 2. Preparation and Properties of Blue Emission CsPbX_3_ PQDs

Fabricating quantum–confined blue emission CsPbX_3_ PQDs with high quality and remarkable stability has been one of the major challenges towards PeLED applications. The most widely used synthetic methods are the hot–injection (HI) method and ligand–assisted reprecipitation (LARP) technique.

### 2.1. Hot–Injection Method

The HI route is a frequently used method for synthesizing both traditional chalcogenide and perovskite colloidal quantum dots (QDs) due to its convenience and simplicity. Protesescu et al. firstly reported the synthesis of monodisperse CsPbX_3_ cubic phase PQDs with size range of 4–15 nm and emission spectral region of 410–700 nm in 2015 [8]. A typical synthetic process was performed in an inert atmosphere that could be summarized as: Cs–oleate precursor was prepared in advance by dissolving CsCO_3_ in octadecene (ODE) with ligand of oleic acid (OA) under an inert gas atmosphere. The cesium precursor was then injected into a lead halide (PbX_2_) solution containing OA, oleylamine (OAm), and ODE at a specific temperature (120–300 °C). After reacting for a few seconds, the reaction mixture was quickly terminated by an ice–water bath. On the basis of the high crystallization kinetics, the reserved PQDs have consolidated size. The crystal size could be regulated by the injection temperature and ligand concentration, adjusting to achieve the wavelength tuning. In addition, the concentration of OA and OAm played a key role in controlling the shape of CsPbBr_3_ PQDs [9].

**Figure 2:**
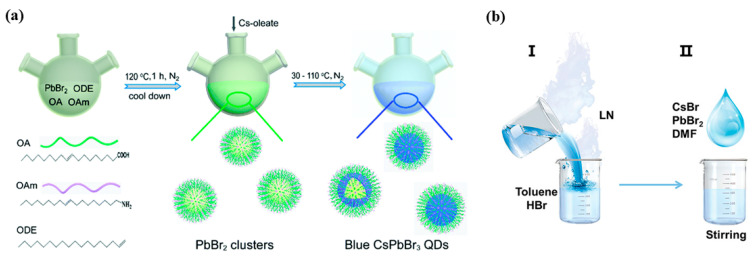
Schematic illustration of hot–injection (HI) method and ligand–assisted reprecipitation (LARP) method. (**a**) The typical synthesis process of PbBr_2_ clusters and CsPbBr_3_ QDs. Reproduced with permission from Ref. [11]. Copyright Royal Society of Chemistry, 2021. (**b**) LN–treated solution–processed CsPbBr_3_ perovskites. Reproduced with permission from Ref. [18]. Copyright Wiley, 2021.

In fact, precise size control and synthesis of ultrasmall PQDs are difficult to achieve due to the ultrafast formation rate within the subsecond scale and soft ionic lattice structures. Relatively large PQDs with edge lengths exceeding 10 nm were normally obtained in previous studies [10]. In this case, flexible bandgap regulation for the larger PQDs is unrealistic due to the weak excitonic confinement regime, and thus Br/Cl anion substitution has been the predominant strategy. As is shown in Figure 2a, Tu et al. obtained ultrasmall blue CsPbBr_3_ PQDs with emission wavelength of 460~497 nm through a modified HI process, in which the pre–synthesized PbBr_2_ clusters were used as nucleation sites to react with Cs–oleate at a relatively low temperature (30–110 °C) [11]. Wang et al. fabricated CsPbBr_3_@amorphous CsPbBr*_x_* core–shell PQDs by the HI method, which showed a significantly higher PLQY of 84% with emission peak at 463.4 nm than that of CsPbBr_3_ QDs (PLQY = 54%) [12]. Akkerman et al. proposed a room–temperature synthesis method for precisely tuning the shape and size of PQDs within a range of 3–13 nm, which enabled temporally separate nucleation and growth processes and thus slowed down the formation rate of PQDs on a time scale of up to 30 min [13].

### 2.2. Ligand–Assisted Reprecipitation Method

The ligand–assisted reprecipitation (LARP) method is also a promising alternative for the preparation of PQDs. LARP is performed by rapidly injecting the precursors that dissolve in a good solvent (polar solvent) into a poor solvent (nonpolar solvent), leading to rapid nucleation and crystal growth under the control of ligands due to the reprecipitation effect. For the first time, Zeng’s group developed a preparation process for CsPbX_3_ PQDs according to supersaturated recrystallization (SR), which could be carried out at room temperature without inert atmosphere [14]. Specifically, the ion sources of CsX and PbX_2_ were firstly well dissolved in solvents such as dimethyl formamide (DMF) or dimethyl sulfoxide (DMSO) in the presence of OA and OAm to form a mixed solution. This mixed solution was then added to toluene that is a solvent with poor solubility for the above ions, resulting in rapid recrystallization and generation of CsPbX_3_ PQDs according to Equation (1). This process could be explained as the huge difference in solubilities between DMF or DMSO and toluene that induced a highly supersaturated state. The size, composition, and optical properties of the CsPbX_3_ PQDs could be controlled by OA/OAm concentration, crystallization temperature, and the ratio of halogen ions. Although operated at room temperature, the as–prepared CsPbX_3_ PQDs still had 70% of PLQY and narrow full–width at half–maximum (FWHM) of 18 nm for blue emission.

Cs^+^ + Pb^2+^ + 3X^−^ → CsPbX_3_ (1)

This facile LARP method has facilitated the large–scale production of PQDs in the atmosphere. Nevertheless, the PQDs obtained with the LARP method showed inferior uniformity compared with that prepared by the HI method, and the PQD–sensitive polar solvents that were employed during the LARP process increased the probability of PQD degradation. Optimizing ligands and removing the polar solvents are critical for the improvement of stability. In order to improve the nucleation control and crystal growth process of PQDs, didodecyl dimethyl ammonium bromide (DDAB) was added to the poor solvents before the injection of precursors, generating deep–blue emission CsPbBr_3_ PQDs with narrow bandwidth and remarkable stability [15]. In addition, a larger molecule, single (6–amino–6–deoxy) beta cyclodextrin, was regarded as both a ligand and confined–growth template to facilitate the synthesis of extremely small CsPbBr_3_ PQDs (1–2 nm) with significant quantum confinement effect and 72.4% of PLQY [16]. Shu and coworkers proposed a two–step supersaturated recrystallization method for blue–emitting CsPbBr_3_ PQDs at room temperature. In detail, a precursor solution containing PbBr_2_, CsBr, OA, OAm, and DMF was firstly prepared, different volumes of toluene were then added to the above precursor in two steps to control the size of QDs, and CsPbBr_3_ PQDs were obtained after use of a 365 nm UV lamp. This method has resulted in extremely small CsPbBr_3_ PQDs of 2.8 nm or less, as well as a high PLQY of 87.20% and a long PL lifetime of 12.24 ns [17]. Cao et al. proposed a cryogenic–temperature synthetic strategy using liquid nitrogen to induce the supersaturation of precursors and then suppress the nucleation and growth rate of PQDs, as shown in Figure 2b. The as–prepared ultrasmall CsPbBr_3_ PQDs (~3 nm) presented deep–blue emission (emission peak at 466 nm) with a narrow FWHM of 12 nm and high PLQY of 98%, indicating that the cryogenic temperature could remove trap states and inhibit nonradiative recombination [18].

## 3. CsPbX_3_ Based Blue–Emitting PeQLEDs

### 3.1. Strategies for Efficiency Enhancement of Blue PeQLEDs

Similar to other types of PeLEDs, the device structures of blue PeQLEDs can be divided into n–i–p or p–i–n architectures. The PQD emission layer is placed between an electron transport layer (ETL) and a hole transport/injection layer (HTL/HIL). In this sandwich structure, electrons and holes enter directly into the perovskite layer through the ETL and HTL, respectively, where they combine into excitons and then generate electroluminescence (EL) emission by radiation recombination. Blue PeQLEDs were firstly reported by Zeng’s group in 2015 with an EQE of 0.07% [6], strenuous efforts were subsequently conducted to improve their performance, as summarized in Table 1. The performance of blue PeQLEDs suffers from lots of limitations involved in efficiency roll–off, short operating lifetime, and considerably lower EQE. Park et al. demonstrated that the luminance efficiency roll–off was mainly attributed to the luminescence quenching caused by nonradiative recombination and hole transfer [19]. In addition, the deeper valence band maximum (VBM) of blue PQDs impedes the hole injection, resulting in imbalanced charge injection and thus poor device performance. A high EQE requires a high PLQY, and increasing radiative and simultaneously decreasing nonradiative recombination rate are needed. Various works have been performed to address the above problems, and the relevant strategies on efficiency enhancement of blue–emitting PeQLEDs will be summarized in the following.

#### 3.1.1. Compositional Engineering

The PL emission range could be readily regulated within the entire visible spectrum range by means of quantum confinement effect and composition engineering. In particular, for the blue emission wavelength below 475 nm, mixed halide CsPb(Br,Cl)_3_ perovskites are commonly needed. Their emission wavelength is tunable through controlling the ratio of Br/Cl, because the halide anion exchange and lattice reconstruction are easily carried out at room temperature considering the high anion mobility [50]. It was revealed that CsPbBr_3_ PQDs possessed higher structural stability and lower trap density than CsPbBr_3−x_Cl_x_ PQDs due to the smaller Cl^−^ ions [19]. The high Cl content in perovskites caused low PLQY because Cl–based perovskites had lower defect tolerance compared to their Br and I counterparts [51]. Additionally, the difference between Cl^−^/Br^−^ radii and binding energy has led to their different migration rates. Consequently, mixed halide devices always suffered from color instability and splitting of EL emission peak, which could be reasonably explained as the electrical bias–driven phase segregation under operating conditions [52].

**Figure 3:**
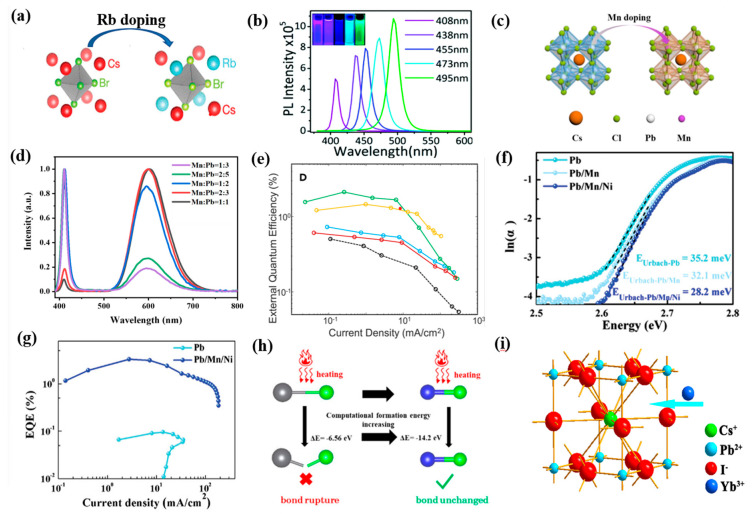
(**a**) Schematic diagram of Rb doping. Reproduced with permission from Ref. [61]. Copyright American Chemical Society, 2019. (**b**) The PL spectra of Eu^3+^–doped Cs*_x_*K_1−*x*_PbCl*_y_*Br_3−*y*_ QDs. Reproduced with permission from Ref. [64]. Copyright Royal Society of Chemistry, 2018. (**c**) Schematic diagram of the Cs(Pb/Mn)Cl_3_; (**d**) PL spectra of Mn: CsPbCl_3_ with different molar ratios of Mn: Pb. Reproduced with permission from Ref. [66]. Copyright Elsevier, 2021. (**e**) The EQE of the fabricated devices. Reproduced with permission from Ref. [24]. Copyright Cell Press, 2018. (**f**) Urbach energies of Pb–, Pb/Mn–, and Pb/Mn/Ni–PQDs; (**g**) EQE–current density characteristics. Reproduced with permission from Ref. [46]. Copyright Wiley, 2022. (**h**) Schematic diagram of the suggested mechanism. Reproduced with permission from Ref. [68]. Copyright American Chemical Society, 2019. (**i**) Structural representation of the CsPbI_3_:Yb^3+^ NCs. Reproduced with permission from Ref. [76]. Copyright Elsevier, 2020.

In view of these problems, several successful approaches have been suggested [53,54,55], in which a doping strategy attracts vast attention whether used to passivate PQDs or promote blueshift of light emission. For the ABX_3_ structure of CsPbX_3_ PQDs, doping–specific ions in the A– and B–site can offer a convenient way to tune the optoelectronic properties and radiative recombination dynamics, and to improve and stabilize emission. A–site substitution was proved to be effective, and cations such as Rb^+^, Ni^2+^, Na^+^ have been employed to partially substitute Cs^+^ [56,57,58,59,60]. As shown in Figure 3a, Rb doping has alleviated the harmful effects of Cl and led to enhanced PLQY. In addition, the absorption of Rb_x_Cs_1−x_PbX_3_ (X = Cl or Br) could be regulated in the wavelength range of 395~525 nm, and more Rb doping caused blueshift of the PL emission peak [61,62,63]. Pan et al. indicated that 2.5% N^2+^ ion–doped CsPbCl_0.99_Br_2.01_ PQDs achieved about a 3–fold increase in PL emission compared with that of undoped PQDs [38]. K^+^ and Eu^3+^ ions were also introduced to obtain Cs*_x_*K_1−*x*_PbCl_3_:Eu^3+^ PQDs as presented in Figure 3b, leading to improved PLQY from the initial 3.2% to 31.2% benefiting from the K–related passivation for the surface of QDs as well as a Eu^3+^–induced decrease in Cl vacancies [64].

B–site doping is also a frequently used method to modulate the stability and optical properties of PQDs, especially partially exchanging Pb^2+^ with isovalent cations [65]. For example, Mn doping could inhibit ion migration and thus improve the emission stability of blue PeQLEDs under operation conditions (Figure 3c). Approaches including one–pot synthesis and post–synthetic anion exchange have been explored for doping Mn^2+^ in CsPbX_3_ QDs. Ma et al. revealed that the Mn:CsPbCl_3_ PQDs were intrinsically stable with <25% or 40–50% Mn concentration (Figure 3d) [66]. A 3–fold increase in PLQY as well as improved EQE were achieved for the Mn–doped CsPb(Br,Cl)_3_ PQDs with pure blue luminescence (Figure 3e) [24]. As shown in Figure 3f,g, Pan et al. reported simultaneously doping Mn and Ni in the B–site to elevate the valence band of CsPb(Br_1.8_Cl_1.2_) PQDs and facilitate hole injection, resulting in an EQE of 3.31% with emission wavelength at 469 nm [46]. Ni^2+^ doping has led to narrower PL emission and the greatly enhanced PLQY of CsPbCl_3_ from 2.4% to 96.5% [67]. Additionally, the Cu–doped CsPb_1−*x*_Cu*_x_*(Br/Cl)_3_ QDs showed better thermal stability and luminescence performance than that of CsPb(Br/Cl)_3_ QDs since the smaller Cu^2+^ caused a lattice contraction and elimination of halogen vacancies (Figure 3h) [68]. Chen and coworkers presented a strategy of co–doping Rb^+^ and Ni^2+^ to effectively passivate the surface defects and decrease the energy barrier for hole injection [33]. Stam et al. doped Sn^2+^, Cd^2+^, and Zn^2+^ into CsPbBr_3_ through a post–synthetic cation exchange process, resulting in lattice contraction and the linear blueshift of optical spectra [69]. Moreover, trivalent lanthanide metal cation (Yb^3+^, Eu^3+^, Gd^3+^, Tm^3+^, or Er^3+^) doping was also performed to tune their photophysical properties and to facilitate a desired luminesce spectrum range [70,71,72,73,74]. For instance, as provided in Figure 3i, an increased PLQY of from 20% to 66.7% and blueshift of emission were achieved after doping Yb^3+^ into CsPbI_3_ PQDs [75,76]. Similarly, Nd^3+^–doped CsPbBr_3_ PQDs achieved excellent stability and an enhanced PLQY with tunable wavelength from 515 to 450 nm, which was explained as the Nd^3+^ doping–induced increase in charge effective masses and oscillator strength [77,78,79]. Other heterovalent metal cations were also doped into CsPbX_3_ PQDs. Al doping was performed by introducing AlBr_3_ precursor during the growth of CsPbBr_3_ to replace Pb^2+^ with Al^3+^, leading to the blueshift of the emission peak from 515 to 456 nm [80]. The similar bond dissociation energy between Al–Br and Pb–Br made it easy for Al^3+^ to bind to the host lattice of perovskite and form dimeric Al_2_Br_6_. Partially replacing Pb^2+^ with Sb^3+^ has driven the blueshift of absorbance and enhanced stability of CsPbBr_3_ NCs due to stronger Sb–Br bonds [81].

#### 3.1.2. Ligand Engineering

**Figure 4:**
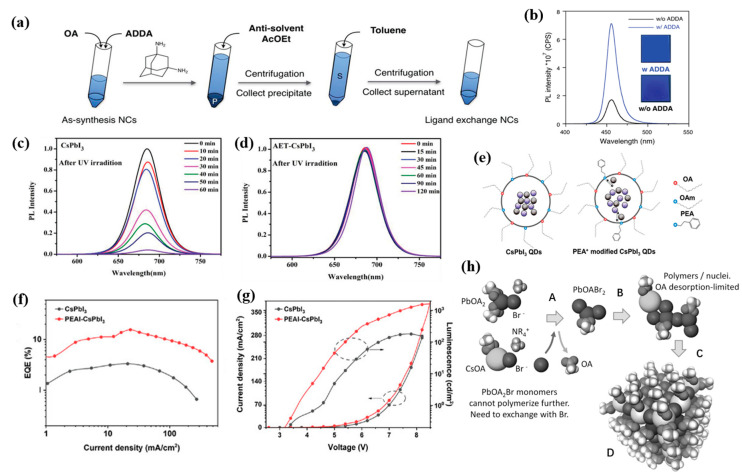
(**a**) Scheme of the ligand exchange and purification process; (**b**) PL spectra of CsPb(Cl/Br)_3_ NC films. Reproduced with permission from Ref. [40]. Copyright 2020, Wiley. Evolution of the PL spectra of (**c**) CsPbI_3_ QDs and (**d**) AET–CsPbI_3_ QDs after UV illumination for different times. Reproduced with permission from Ref. [85]. Copyright 2019, Wiley. (**e**) Schematic diagram of the effects of PEA^+^ ligands on carrier behavior in the QDs; (**f**) EQE–*J* curves of the LEDs based on the pristine CsPbI_3_ QDs and PEAI−CsPbI_3_ QDs; (**g**) *J*–*V*–*L* curves of the LEDs based on the pristine CsPbI_3_ QDs and PEAI–CsPbI_3_ QDs. Reproduced with permission from Ref. [86]. Copyright 2022, Wiley. (**h**) Density functional theory molecular dynamics simulation of CsPbBr_3_ nucleation and growth steps. Reproduced with permission from Ref. [21]. Copyright 2016, Wiley.

PeQLEDs typically exhibit tight links between good PL and poor EL properties. The long–chain ligands around PQDs enable the good monodisperse stability and benefit their high PLQYs. However, insulating long–chain ligands is not conducive to charge transport within PQD films, even leading to high turn–on voltage and poor device performance [82]. The ligand–induced “hopping” transport mode closely depends on the ligand type and interparticle distance [83]. Long–chain ligands such as OAm and OA are generally used for synthesizing and stabilizing the PQDs, which also interrupt the charge transport between each of PQDs. With this in mind, ligand exchange has been performed to replace the OA or OAm ligands with shorter ligands. For example, short ligands of acetate and butylammonium (BA) have resulted in denser passivation for PQDs and better stability [84]. As presented in Figure 4a, the ligand–exchanged CsPb(Cl/Br)_3_ with adamantane–1,3–diamine (ADDA) exhibited blue emission at 456 nm and an EQE of 0.49%, as well as improved PLQY from 11.6% to 31.8% (Figure 4b) [40].

Tian’s group have reported a series of studies on ligand engineering for PQDs. As shown in Figure 4c,d, partially replacing OA and OAm with 2–aminoethanethiol (AET) has enhanced stability and carrier mobility, and passivated the traps of PQDs [85]. They also proposed a hydrogen bromide etching–driven ligand exchange strategy, in which didodecylamine and phenethylamine were introduced as short ligands to achieve stable and pure–blue–emitting CsPbBr_3_ PeQLEDs with a high luminance of 3850 cd/m^2^ [41]. Recently, they employed shorter phenethylammonium (PEA^+^) conjugated ligands to partially replace OAm at the surface of CsPbI_3_ QDs (Figure 4e–g), which facilitated improved hole transport, more balanced charge injection, and low efficiency roll–off [86]. KI and NaI as inorganic metal ligands have been introduced into the mildly polar antisolvent during the PQDs purification process, which effectively reduced the surface traps and improved the stability of PQD films [87]. Chen et al. indicated that employing octanoic acid and octylamine as co–capping ligands could obtain more stable α–CsPbI_3_ PQDs with an enhanced charge transport rate [88]. Other ligand modification methods by introducing 2–hexyldecanoic (DA) [89], amino acids [90], conjugated alkylamine, 3–phenly–2–propen–1–amine (PPA) [91], di–dodecyl dimethyl ammonium halide (DDAX, X = Br/Cl) [25,26,92], sulfobetaine [93] and phosphocholine [94], L–phenylalanine (L–PHE) [95], and propane–1,3–diammonium bromide (PDAB) [96] were also studied. In addition, it was revealed that the proton transfer between OA and OAm could lead to facile ligand loss and poor stability, as shown in Figure 4h, and an OA ligand–only synthetic method was consequently proposed using quaternary alkylammonium halides as precursors [21]. In particular, Cheng et al. in situ synthesized ligand–free PQDs into polyacrylonitrile nanofiber films, achieving a high PLQY of 71% and tunable emissive peaks (448–600 nm) [97].

#### 3.1.3. Surface/Interface Engineering

**Figure 5:**
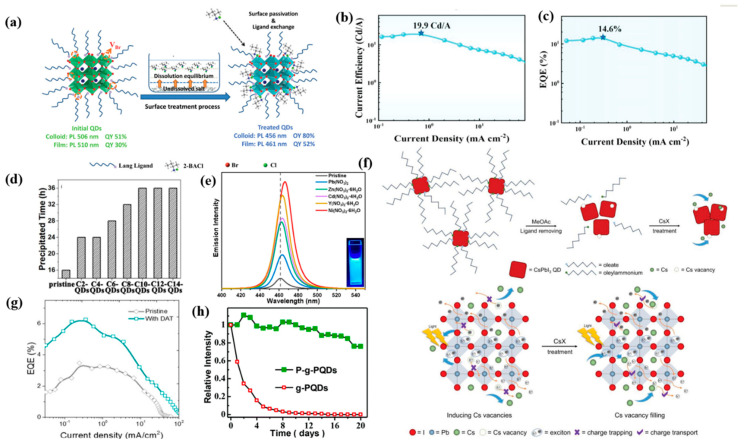
(**a**) Schematic of the surface regulation process. Reproduced with permission from Ref. [98]. Copyright Wiley, 2020. (**b**) The curve of the current efficiency of the device; (**c**) EQE of the devices based on the exchange QDs. Reproduced with permission from Ref. [48]. Copyright Wiley, 2022. (**d**) The precipitation times of the hexane solutions of all the samples. Reproduced with permission from Ref. [99]. Copyright Elsevier, 2021. (**e**) PL spectra. Reproduced with permission from Ref. [101]. Copyright American Chemical Society, 2019. (**f**) Schematic illustrations of CsPbI_3_ QD film deposition and CsX post–treatment process. Reproduced with permission from Ref. [102]. Copyright Wiley, 2019. (**g**) EQE–current density. Reproduced with permission from Ref. [31]. Copyright American Chemical Society, 2020. (**h**) Stability tests of g–PQDs and P–g–PQDs in air. Reproduced with permission from Ref. [104]. Copyright Royal Society of Chemistry, 2019.

Controlling the surface states of PQDs is important for improving charge injection efficiency and reducing trap density. Halogen vacancies can form deep–defect states in perovskites, which will capture charge carriers and provide nonradiative recombination channels. Post–synthesis halide exchange treatments have been proven to diminish the surface trap density and reconstruct the Br/Cl ratio of PQDs. When adding organic ammonium chlorides during the purification process of CsPbBr_3_ PQDs, extra Cl^−^ could fill the Br vacancy at the surface of PQDs and form passivated mixed–halide surfaces. Meanwhile, as shown in Figure 5a, the short–chain ammonium moiety replaced the original long ligands, resulting in a modified PLQY of 80% at 456 nm [98]. Baek et al. achieved improved thermal stability and significantly increased PLQY from 50% to 84–100% after introducing zinc halide and trioctylphosphine oxide (TOPO) as surface capping agents for CsPbBr_3_ QDs [30]. Similarly, Sun et al. employed both InX_3_ (X = Cl, Br, I) and TOPO as surface passivation agents for CsPbX_3_ PQDs, achieving more than 90% PLQY and narrow emission (14–35 nm), as well as high thermal stability [42]. The synergy effect of InX_3_–TOPO has effectively inhibited the halogen vacancies and surface defects, and reduced the Pb–O bond in the crystal lattice. The enriched–bromine surface state of Cd–doped CsPbBr_x_Cl_3−x_ QDs was realized by anion exchange using a PbBr_2_ stock solution, leading to efficient surface passivation and improved PLQY and lifetime. At the same time, significantly enhanced carrier transport capability was achieved after replacing the long–chain OA/OAm with PbBr_2_. High–performance sky–blue PeLEDs at 490 nm were obtained with a fascinating EQE of 14.6% and current efficiency of 19.9 cd/A (Figure 5b,c) [48].

Generally, adjusting the proportion of halogens makes it easy to obtain blue PQDs, however, precisely adjusting the halide ratio of mixed Br/Cl perovskites limits the passivation of Cl vacancies, resulting in poor device performance. Therefore, developing new passivation substances without halogen is also meaningful. As shown in Figure 5d, hydrophobic ionic liquids were used to passivate CsPbX_3_ QDs due to their stronger interaction with the Pb^2+^ on the surface compared with the initial OA/OAm, and the longer alkyl chain promoted better QD stability [99]. KSCN was used as passivating material for CsPbBr_x_Cl_3−x_ PQDs, both K^+^ and SCN^−^ ions could fill the defects in PQDs, and K^+^ confined the halide anions and thus prevented halide migration and structural degradation [100]. Nitrate anion treatment was also extremely effective for selectively eliminating the surface defects of PQDs, and employing Ni(NO)_3_·6H_2_O has facilitated an impressive PLQY of 85%, as shown in Figure 5e [101]. Concretely, the nitrate ions in PQD/toluene solution could desorb the undesired metallic lead and combine with excess surface metal ions, while maintaining the crystal structure and surface ligands of perovskites. As is shown in Figure 5f, Ma’s group proposed passivating CsPbI_3_ PQD films through a Cs–salt post–treatment with cesium acetate (CsAc), cesium carbonate (CsCO_3_), and cesium nitrate (CsNO_3_), respectively, resulting in enhanced electron coupling between PQDs and reduced surface vacancies [102]. Zheng et al. used n–dodecyl ammonium thiocyanate (DAT) to passivate Cl vacancies, in which thiocyanate (SCN^−^) groups filled Cl spaces and removed electron traps within band gaps, resulting in twice the EQE (Figure 5g) [31].

Additionally, surface/interface passivation can also improve the water resistance and stability of PQDs in the atmospheric environment. Recently, the stability of PQDs has been improved by organic and inorganic modification of the PQD surface. Metal oxide coatings such as SiO_2_, ZnS, and Al_2_O_3_ have been deposited onto the surface of PQDs to improve their resistance to moisture and meanwhile to prevent anion exchange effects. For example, fabricating SiO_2_ coating on the surface of CsPbX_3_ QDs has been verified to enhance QD stability through forming a cross–linked silica network. CsPb(Br/Cl)_3_ PQDs with an Al–doped CdSe layer also presented remarkable stability under thermal exposure, water, and UV irradiation [103]. Similarly, encapsulating PQDs in hydrophobic polymer matrices such as solid paraffin also realized enhancement of light and water stabilities in atmosphere (Figure 5h) [104].

#### 3.1.4. Device Structural Engineering

**Figure 6:**
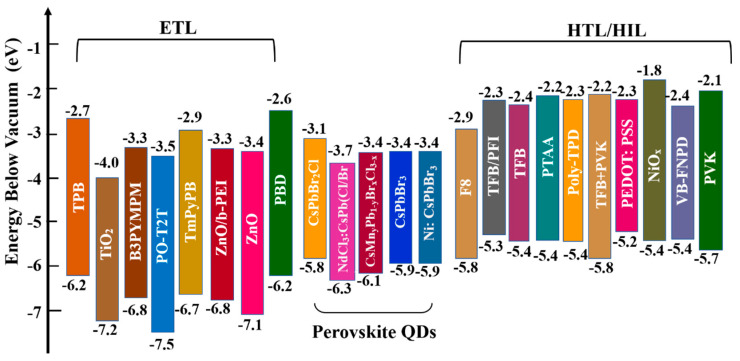
Energy diagram of the commonly used ETL, PQD, HTL/HIL materials in blue PQD–based LEDs.

Establishing suitable energy–level alignment between adjacent layers to reduce energy barriers and realize balanced charge injection is important for device performance. Some representative ETL, PQD, and HTL/HIL materials reported for blue PeQLEDs are presented in Figure 6. The widely used organic HTL materials include poly(9–vinlycarbazole) (PVK), poly (N,N′–bis(4–butylphenyl)–N,N′–bis(phenyl)–benzidine) (poly–TPD), and poly(9,9–dioctylfluorene–co–N–(4–(3–methylpropyl))diphenylamine) (TFB). In addition, several metal oxides with excellent stability and suitable energy levels, including WO*_x_*, MoO*_x_*, NiO*_x_*, Cu*_x_*O, and VO*_x_*, also serve as HTL/HIL. For electron transport, ZnO is the most used ETL in blue PeLEDs due to its high mobility, low electron injection barrier, and effective hole blocking. Generally, the electron mobility of ETL is 10^−3^–10^−4^ cm^2^ V^−1^s^−1^, which is much higher than the hole mobility of HTL (10^−4^–10^−6^ cm^2^ V^−1^s^−1^), resulting in unbalanced charge injection and carrier accumulation [105]. Enhancing hole mobility and lowering hole injection barriers to alleviate the carrier accumulation and reduce exciton quenching are urgently needed for PeQLEDs.

**Figure 7:**
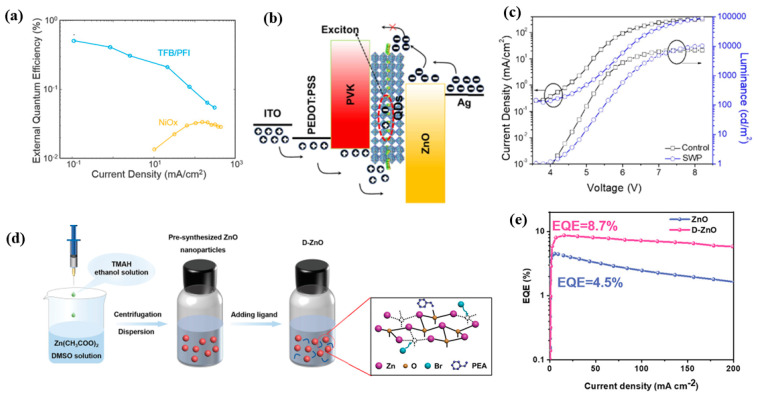
(**a**) EQE curves for LEDs fabricated using 469 nm perovskite nanocrystals and either NiO_x_ (orange) or TFB/PFI (blue) as the HTL. Reproduced with permission from Ref. [23]. Copyright Wiley, 2018. (**b**) The energy level alignment with the proposed emission mechanism for the PeLEDs based on SWP QDs films; (**c**) current density and luminance of the PeLEDs versus bias. Reproduced with permission from Ref. [45]. Copyright Elsevier, 2022. (**d**) Schematic synthesis diagram of the D–ZnO colloidal nanocrystal solution; (**e**) the EQE of the champion PeLEDs based on ZnO and D–ZnO ETL. Reproduced with permission from Ref. [49]. Copyright Wiley, 2022.

Improved hole transport and injection have been realized through introducing dopants in the current HTLs to increase mobility, or employing a bilayer HTL structure to take advantage of the high hole mobility of poly–TPD and the highest occupied molecular orbital (HOMO) level of PVK [106]. Gangishetty et al. constructed a bilayer TFB/PFI HTL in blue PeLEDs, which incorporated both the high hole mobility and electron–blocking effect of TFB as well as the hole injection of PFI. They indicated that the PFI–induced strong surface dipole caused a band bending of the HTL to a higher work function (Figure 7a), and thus facilitated better hole injection and suppressed nonradiative recombination [23]. Baek et al. used a new cross–linkable VB–FNPD material as the HTL because of its higher hole mobility compared to the conventional PVK HTL [30].

The precise degradation mechanism of blue QLEDs has been investigated. It was revealed that the electron transfer from QDs to ZnO ETL caused operating–voltage rise and charge accumulation in the ETL, resulting in fast degradation at the QD–ZnO junction and short device operating lifetime [107]. Regulating the bandgap and work function of ZnO ETLs by doping or modifying the ZnO itself and inserting an insulating layer (Al_2_O_3_, PVK, PMMA, etc.) at the ETL/QD interface have proved to be effective to delay electron injection and balance charge transfer in devices [108,109]. As shown in Figure 7b,c, Tian’s group inserted a ZnCl_2_ barrier layer between two CsPbBr_3_ QD layers, which not only drove the carrier recombination region away from the QD/HTL interface, but also reduced the electron mobility to balance the charge injection. The resultant pure–blue PeQLEDs achieved the maximum luminance of 10,410 cd m^−2^ and striking operational stability [45]. Recently, they proposed employing the difunctional ZnO (D–ZnO) nanocrystals for device fabrication (Figure 7d). It was revealed that the ligand of D–ZnO could fill the O_v_ defects of ZnO and repair the solvent destroyed by the D–ZnO solution, meanwhile increasing the energy barrier of electron injection. This approach has enabled the pure–blue PeQLEDs to obtain the highest EQE of 8.7% and a maximum luminance of 11,100 cd m^−2^ (Figure 7e) [49].

### 3.2. Enhancement of Device Stability

As with other types of PeLEDs, the origins of the instability of PeQLEDs can be approached from two aspects, namely, the intrinsic chemical inability of PQDs and the unsatisfactory carrier transport in the constituent layers of devices. The weak ionic bonds in perovskites are easily broken by extrinsic factors including electric field, oxygen, moisture, or thermal heating. Moreover, the intrinsic ionic defects such as the halogen vacancies and dangling bonds on the surface of PQDs may evolve into ion migration channels under operating conditions. Previous studies have revealed that the performance degradation and spectral shift for halogen–perovskite–based LEDs is strongly related to the defect–induced ion migration and phase separation, which is typically more notable in the Br/Cl mixed blue emission perovskites [110]. Even though organic ligands on the surface of PQDs effectively hinder the ion migration, performance degradation still the major limiting factor for PeLED application. One reason is that the widely used organic ligands such as OA or OAm may be desorbed from the surface of PQDs and thus generate more surface defects, accelerating the phase transition and degradation of PQDs. As discussed above, metal doping, surface passivation, constructing a core–shell structure, and ligand tailoring have been effective approaches to reduce the defect density in PQDs, and thus prolong the stability of perovskite crystal structure and phase.

Additionally, the operation instability and efficiency roll–off have been attributed to the thermal degradation of perovskite and imbalanced charge injection at high current density [111]. Significantly faster mobility of electrons than holes causes charge accumulation at the perovskite/transport layer interface, deteriorating the device performance. Therefore, fabricating a uniform and dense PQD emitting layer with low defect density, screening chemically stable transport materials, and establishing reasonably arranged multilayers to modulate the charge transport are anticipated solutions to prolong device lifetime. In addition, optimizing device structure to improve the thermal conductivity at high current density is also helpful to operation stability.

## 4. Conclusions and Outlooks

In the past five years, blue–emitting PeQLEDs have experienced some gratifying progress with the increase in EQEs from 2% to 14.6%. However, further performance enhancement, especially for deep–blue and pure–blue emission devices, is still urgent for the display application of full–color PeQLEDs. To summarize, the major obstacles for current blue PeQLEDs can be attributed to the following factors: severe vacancies or defect–mediated nonradiative recombination losses, imbalanced charge injection, unsatisfactory charge transport within the PQD emission layer, as well as the efficiency roll–off and spectral instability of devices under operating conditions.

The previous studies have revealed that the efficiency roll–off is mainly assigned to Joule heating and imbalanced charge injection. Efforts are still needed to develop new suitable HTL/HIL, ETL, and charge blocking materials or improve existing materials, so as to construct a better matched energy level arrangement between functional layers in devices, eventually facilitating balanced charge injection and blocking charge leakage. Appropriate doping is useful for tuning the electron and hole current, and thus balancing charge injection.

The spectral instability issue driven by electric fields still needs to be addressed. To date, multiple A–cation strategies, hydrogen–bonded amine–group doping strategy, metal halide treatment, and anchoring ligands or some insulating polymers onto the PQD surface have been recognized to be effective for increasing the ion migration barrier and passivating surface–related defects [112]. Notably, Ighodalo et al. reported that no ion migration and phase segregation occurred in both the as–prepared CsSn(I_x_Br_1−x_)_3_ and CsSn_y_Pb_1−y_(I_x_Br_1−x_)_3_ (0 < x < 1, y ≥ 0.6) perovskites under either light illumination or a strong electric field. They attributed this situation to the stronger tin–halide bond and thus higher ion migration activation energy compared to the lead–halide counterparts [113]. This study shows again that metallic doping is a promising strategy to inhibit ion migration and, meanwhile, provides us guidance for the design of intrinsically stable perovskites. Moreover, more effective post–treatment and defect passivation approaches are also needed to fill halogen vacancies and reduce defect density. To alleviate the problem of phase segregation, more approaches are imperative to improve the preparation of mixed–halide perovskites so as to achieve uniform distribution of halogen components. For instance, the anion exchange post–treatment process is generally considered to be more convenient for Cl doping and tuning emission wavelength than directly adding Cl^−^ in precursors [114].

Additionally, ligands always play a vital role in suppressing ion migration, maintaining the quantum confinement effects of perovskite layers, and passivating the uncoordinated metal (Pb^2+^, Cs^2+^) defects. However, at the same time, they also bring a series of negative effects including the following aspects which still should be taken into account in the future ligand engineering: 1) the insulating property of most current ligands causes unsatisfactory carrier transport within perovskite layers; 2) excessive ligands will cause a drop in PLQY due to strong electron–photon coupling, which is more serious in Br/Cl mixed–halide devices due to the higher excitonic state of the Cl–doped perovskite layer [115]; 3) desorption of the weakly bonded ligands easily happens and leads to the quenching of PQDs. Further screening of short ligands with passivation groups and suitable triplet energy is an universal approach for impeding triplet and deep trap–assisted nonradiative pathways, as well as improving the charge carrier transport within PQD layers [116]. Tailoring new organic semiconducting ligands and employing some short metal ligands have been demonstrated to be useful for achieving better carrier transport.

## Figures and Tables

**Table 1:** The key parameters of currently reported blue PeQLEDs.

Composition	Device Structure	EL [nm]	EQE [%]	Luminance [cd m^−2^]	*LT*_50_	Year	Ref.
CsPbX_3_	ITO/PEDOS:PSS/PVK/PQDs/TPBi/LiF/Al	455	0.07	742	–	2015	[6]
CsPbBr_x_I_3−x_	ITO/PEDOT:PSS/PVK/PQDs/TPBi/LiF/Al	490	1.9	35	–	2016	[20]
CsPbBr_x_Cl_3−x_	ITO/TiO_2_/PQDs/F8/MoO_3_/Au	495	0.075	–	–	2016	[21]
CsPbBr*_x_*Cl_3−*x*_	ITO/NiO_x_/PQDs/TPBi/LiF/Au	470	0.07	350	–	2017	[22]
CsPbBr*_x_*Cl_3−*x*_	ITO/PEDOT:PSS/TFB/PFI/PQDs/TPBi/LiF/Al	488	1.41	830	–	2018	[23]
CsPbBr*_x_*Cl_3−*x*_	ITO/PEDOT:PSS/TFB/PFI/PQDs/TPBi/LiF/Al	469	0.50	111	–	2018	[23]
CsMn_y_Pb_1−y_Br*_x_*Cl_3−*x*_	ITO/PEDOT:PSS/TFB/PFI/PQDs/TPBi/LiF/Al	466	2.12	245	–	2018	[24]
CsMn_y_Pb_1−y_Br*_x_*Cl_3−*x*_	ITO/PEDOT:PSS/TFB/PFI/PQDs/TPBi/LiF/Al	470	1.46	389	–	2018	[24]
CsPb(Br_x_Cl_1−x_)_3_	ITO/PEDOT:PSS/p–TPD/PVK/PQDs/B3PYMPM/TPBi/LiF/Al	476	2.25	678	–	2019	[25]
CsPb(Br_x_Cl_1−x_)_3_	ITO/PEDOT:PSS/p–TPD/PVK/PQDs/B3PYMPM/TPBi/LiF/Al	490	3.5	2063	0.43 min @100 cd m^−2^	2019	[25]
CsPbBr_x_Cl_3−x_	ITO/PEDOT:PSS/Poly–TPD/PQDs/TPBi/LiF/Al	479	0.864	29.948	0.83 min @0.05 cd m^−2^	2019	[26]
CsPb(Br_x_Cl_1−x_)_3_	ITO/PEDOT:PSS/Poly–TPD/CBP/PQDs/B3PYMPM/LiF/Al	463	1	318	–	2019	[27]
CsPbBr_1.3_Cl_1.7_	ITO/PEDOT:PSS/Poly–TPD/PQDs/TPBi/LiF/Al	461	0.8	763	–	2019	[28]
Ni: CsPbCl_1.7_Br_1.3_	ITO/PEDOT:PSS/Poly–TPD/PQDs/TPBi/LiF/Al	460	1.35	33	0.86 min @0.2 cd m^−2^	2019	[29]
CsPb(Br_x_Cl_1−x_)_3_	ITO/PEDOT:PSS/ PVK/PQDs/ TPBi/LiF/Al	446	0.27	33	–	2020	[30]
CsPb(Br_x_Cl_1−x_)_3_	ITO/PEDOT:PSS/VB–FNPD/PQDs/TPBi/LiF/Al	446	0.56	46	5.4 min @1 cd m^−2^	2020	[30]
CsPb(Br_x_Cl_1−x_)_3_	ITO/TFB/PFI/PQDs/3TPYMB/Liq/Al	471	6.3	465	1.65 min @80 cd m^−2^	2020	[31]
CsPb(Br/Cl)_3_	ITO/PEDOT:PSS/poly–TPD/PQDs/PO–T2T/LiF/Al	477	1.96	86.95	4.5 min @26.66 cd m^−2^	2020	[32]
Rb/Ni:CsPbBr_1.8_Cl_1.2_	ITO/TEDOT:PSS/Poly–TPD/PVK/PQDs//TPBi/LiF/Al	467	2.14	–	–	2020	[33]
CsPbBr_3_	ITO/PEDOT:PSS/PTAA/PQDs/TPBi/LiF/Al	479	12.3	90	20 min @90 cd m^−2^	2020	[34]
CsPbBr_x_Cl_3−x_	ITO/PEDOT:PSS/Poly–TPD/PQDs/TPBi/Ca/Ag	469	0.65	30	–	2020	[35]
CsPbBr_x_Cl_3−x_	ITO/PEDOT:PSS/Poly–TPD/PQDs/TPBi/Ca/Ag	479	1.0	119	–	2020	[35]
CsPbBr_x_Cl_3−x_	ITO/PEDOT:PSS/Poly–TPD/PQDs/TPBi/Ca/Ag	489	1.8	182	–	2020	[35]
CsPbBr_x_Cl_3−x_	ITO/PEDOT:PSS/Poly–TPD/PQDs/TPBi/Ca/Ag	496	2.6	603	–	2020	[35]
La:CsPb(Br_x_/Cl_1_*_−__x_*)_3_	ITO/PEDOT:PSS/PVK/PQDs/TPBi/LiF/Al	480	2.17	292.7	–	2020	[36]
La:CsPb(Br_x_/Cl_1_*_−__x_*)_3_	ITO/PEDOT:PSS/PVK/PQDs/TPBi/LiF/Al	489	3.25	192.6	–	2020	[36]
NdCl_3_:CsPbBr_3_	ITO/PEDOT:PSS/TFB/PQDs/TPBi/Liq/Al	478	2.7	138	0.2 min @20 cd m^−2^	2020	[37]
Ni:CsPbCl_x_Br_3−x_	ITO/PEDOT:PSS/TFB/PFI/PQDs/TPBi/LiF/Al	470	2.4	612	–	2020	[38]
CsPb(Br_x_Cl_1−x_)_3_	ITO/PEDOT:PSS/poly–TPD/PVK/PQDs/TmPyPB/LiF/Al	470	2.15	507	0.39 min @232.4 cd m^−2^	2020	[39]
CsPb(Br_x_Cl_1−x_)_3_	ITO/PEDOT:PSS/TFB/PQDs/TPBi/Liq/Al	456	1.1	43.2	0.083 min @10 cd m^−2^	2020	[40]
CsPbBr_3_	ITO/PEDOS:PSS/PVK/PQDs/ZnO/Ag	470	4.7	3850	720 min @102 cd m^−2^	2021	[41]
CsPbBr_3_	ITO/NiO_x_/TFB/InX–TOPO–PQDs/PBD/ZnO/Al	463	1.62	164	–	2021	[42]
CsPbBr_3_	ITO/NiO_x_/TFB/InX–PQDs/PBD/ZnO/Al	465	0.38	41	–	2021	[42]
CsPbBr_x_Cl_3−x_	IFO/ZnO/b–PEI/PQDs/PVK/V_2_O_5_/Al	492	0.053	143.1	–	2021	[19]
Cs_4_PbBr_6_/CsPbBr_3_	ITO/PEDOT:PSS/TPB + PVK/PQDs/TPBi/LiF/Al	480	4.65	23	1.5 min @75 cd m^−2^	2021	[43]
CsPbCl_x_Br_3−x_	ITO/PEDOT:PSS/poly–TPD/PQDs/TPBi/Ca/Ag	482	0.99	177	–	2021	[44]
AMCl:CsPbCl_x_Br_3−x_	ITO/PEDOT:PSS/poly–TPD/PQDs/TPBi/Ca/Ag	481	1.53	193	–	2021	[44]
PMCl:CsPbCl_x_Br_3−x_	ITO/PEDOT:PSS/poly–TPD/PQDs/TPBi/Ca/Ag	477	1.31	175	–	2021	[44]
BMCl:CsPbCl_x_Br_3−x_	ITO/PEDOT:PSS/poly–TPD/PQDs/TPBi/Ca/Ag	479	2.30	205	–	2021	[44]
SWP:CsPbBr_3_	ITO/PEDOS:PSS/PVK/PQDs/PQDs/ZnO/Ag	469	5	10,410	80 min @1700 cd m^−2^	2022	[45]
CsMnNiPbBr_3_	ITO/PEDOS:PSS/poly–TPD/PVK/PQDs/TPBi/LiF/Al	463	3.31	60.2	8 min @60.2 cd m^−2^	2022	[46]
PEAX:CsPbBr_3_	ITO/PEDOT:PSS/poly–TPD/TFB/PVK/PQDs/TPBi/LiF/Al	489	1.81	456	0.8 min @100 cd m^−2^	2022	[47]
Cd:CsPb(Br_x_Cl_1−x_)_3_	ITO/PEDOT:PSS/poly–TPD/PQDs/TPBi/LiF/Al	490	14.6	403	12 min @134 cd m^−2^	2022	[48]
CsPbBr_3_	ITO/PEDOS:PSS/PVK/PQDs/D–ZnO/Ag	470	4.5	7817	360 min @100 cd m^−2^	2022	[49]
CsPbBr_3_	ITO/PEDOS:PSS/PVK/PQDs/ZnO/Ag	470	8.7	11,100	2100 min @100 cd m^−2^	2022	[49]

## Data Availability

The data presented in this review are available on request from the corresponding author.
